# Personalized Neoantigen Vaccines Synergize with Immune Checkpoint Therapy and CD8-Targeted Cytokines to Control B-Cell Lymphoma

**DOI:** 10.64898/2026.08.02.742304

**Published:** 2026-08-06

**Authors:** Yuang Song, Ekaterina Aladyeva, Ruan F. Vieira Medrano, Derek J Theisen, Cora D. Arthur, J Michael White, Heather Brink Kohlmiller, Anthony Vomund, Kartik Singhal, My Hoang, Samuel Ameh, Kathleen C.F. Sheehan, Ronald Levy, Todd A. Fehniger, Maxim N. Artyomov, Malachi Griffith, Obi L. Griffith, Yik Andy Yeung, Ivana Djuretic, Hussein Sultan, Robert D. Schreiber

## Abstract

Personalized neoantigen (neoAg) vaccines have shown clinical promise in solid tumors ^1–8^ , yet their efficacy and mechanism of action in hematopoietic malignancies remain poorly defined ^9–11^ . Herein, we establish an immunocompetent syngeneic A20 B-cell lymphoma platform to test the efficacy of neoAg vaccines used either as mono- or combinatorial therapies with other immunotherapies ^12–17^ . Whereas subcutaneous A20 tumors were refractory to single-agent αPD-1 or αCTLA4 therapy, they were eradicated in a T cell-dependent manner in 90% of syngeneic hosts treated with dual immune checkpoint therapy (dual ICT, i.e., αPD-1 + αCTLA4). By mapping antigen specificity of dual-ICT-elicited T cells, we identified and validated dominant endogenous A20 MHC-I and MHC-II neoantigens and designed therapeutic synthetic long peptide (SLP) vaccines containing these neoepitopes. This vaccine (A20 neoVAX) promoted robust neoAg-specific CD4□ and CD8□ T cell responses in naïve syngeneic BALB/c mice and induced tumor rejection in ∼70% of subcutaneous tumor-bearing mice. In addition, nearly all mice rejected their subcutaneous A20 tumors when A20 neoVAX was combined with αPD-1. To render the results of this study more physiologic, we developed a systemic A20 lymphoma model and found that dual ICT failed to control tumor progression and A20 neoVAX delayed tumor progression and prolonged animal survival but did not induce tumor rejection. In contrast, A20 neoVAX plus dual ICT achieved durable systemic tumor elimination. Mechanistically, the combination of A20 neoVAX plus dual ICT amplified priming of A20 neoAg-specific T cells, prevented T cell dysfunction, sustained the cytotoxic capacity of tumor-specific CD8 ^+^ T cells, and induced Th1-skewing of CD4 ^+^ T cells in tumor and peripheral compartments. To increase the clinical relevance of these findings and to minimize potential adverse events in tumor-bearing, therapeutically treated individuals, we substituted CD8-targeted cytokine muteins (CD8-IL2 or CD8-IL21) for αCTLA4. These agents represent genetically modified forms of IL-2 or IL-21 that selectively stimulate CD8 ^+^ T cells but have significantly reduced capacity to activate chronic inflammation and immunosuppressive functions of other immune cells. Whereas mice bearing systemic A20 lymphoma treated with either nothing, A20 neoVAX, or A20 neoVAX + CD8-IL2 failed to control tumor outgrowth, 66.7% of tumor-bearing mice treated with A20 neoVAX + CD8-IL2 + αPD-1 rejected their tumors. In similar experiments in which CD8-IL21 was substituted for CD8-IL2, tumor clearance was also observed in two-thirds of A20-bearing mice but now rejection occurred in the absence of αPD1. Together, these data define a framework for optimal personalized neoAg vaccination in B-lymphoma and demonstrate that neoAg vaccines can safely synergize with CD8 ^+^ T cell-selective immunotherapies to prevent T-cell dysfunction and generate durable systemic anti-tumor immunity.

## Full Text Availability

The license terms selected by the author(s) for this preprint version do not permit archiving in PMC. The full text is available from the preprint server.

